# Vitamin and antioxidant rich diet increases *MLH1* promoter DNA methylation in DMT2 subjects

**DOI:** 10.1186/1868-7083-4-19

**Published:** 2012-10-01

**Authors:** Olivier J Switzeny, Elisabeth Müllner, Karl-Heinz Wagner, Helmut Brath, Eva Aumüller, Alexander G Haslberger

**Affiliations:** 1 Department of Nutritional Sciences, University of Vienna, Althanstraße 14, Vienna, 1090, Austria; 2 Diabetes Outpatient Clinics, Health Centre South Vienna, Wienerbergstraße 13, Vienna, 1100, Austria

**Keywords:** MLH1, ROS, DNA methylation, Demethylation, Nutritional intervention, Diabetes, Antioxidant, Pyrosequencing

## Abstract

**Background:**

Oxidative stress may lead to an increased level of unrepaired cellular DNA damage, which is discussed as one risk for tumor initiation. Mismatch repair (MMR) enzymes act as proofreading complexes that maintain the genomic integrity and MMR-deficient cells show an increased mutation rate. One important gene in the MMR complex is the MutL homolog 1 (*MLH1*) gene. Since a diet rich in antioxidants has the potential to counteract harmful effects by reactive oxygen species (ROS), we investigated the impact of an antioxidant, folate, and vitamin rich diet on the epigenetic pattern of *MLH1*. These effects were analyzed in individuals with non-insulin depended diabetes mellitus type 2 (NIDDM2) and impaired fasting glucose (IFG).

**Methods:**

In this post-hoc analysis of a randomized trial we analyzed DNA methylation of *MLH1*, *MSH2*, and *MGMT* at baseline and after 8 weeks of intervention, consisting of 300 g vegetables and 25 ml plant oil rich in polyunsaturated fatty acids per day. DNA methylation was quantified using combined bisulfite restriction enzyme analysis (COBRA) and pyrosequencing. *MLH1* and *DNMT1* mRNA expression were investigated by qRT-PCR. DNA damage was assessed by COMET assay. Student’s two-tailed paired t test and one-way ANOVA with Scheffé corrected Post hoc test was used to determine significant methylation and expression differences. Two-tailed Pearson test was used to determine correlations between methylation level, gene expression, and DNA strand break amount.

**Results:**

The intervention resulted in significantly higher CpG methylation in two particular *MLH1* promoter regions and the *MGMT* promoter. DNA strand breaks and methylation levels correlated significantly. The expression of *MLH1*, *DNMT1*, and the promoter methylation of *MSH2* remained stable. CpG methylation levels and gene expression did not correlate.

**Conclusion:**

This vitamin and antioxidant rich diet affected the CpG methylation of *MLH1*. The higher methylation might be a result of the ROS scavenging antioxidant rich diet, leading to lower activity of DNA demethylating enzymes. Our results suggest the hypothesis of CpG demethylation via DNA repair enzymes under these circumstances. NIDDM2 and IFG patients benefit from this simple dietary intervention involving epigenetic and DNA repair mechanisms.

## Background

Diabetes mellitus type 2 (T2DM) is a multifactorial disease characterized by hyperlipidemia, visceral obesity, hypercoagulability, microalbuminuria and hypertension based on genetic predisposition and environmental factors resulting in insulin resistance and hyperglycemia. Diabetic patients have often been described as being under enhanced oxidative stress [1,2]. Long-term dietary patterns and status have a large impact on the risk developing non-communicable diseases like T2DM. Chronic exposure to elevated amounts in particular of free fatty acids and palmitate (C_16_ saturated fatty acid) leads to a higher reactive oxygen species (ROS) burden, while long chain polyunsaturated fatty acids such as docosahexaenoic acid (DHA) has the opposite effect [3,4]. The phenotype of chronic hyperglycemia leads to increased production of ROS also originating from the substrate overwhelmed electron transport system in the mitochondria and the plasma membrane nicotinamide adenine dinucleotide phosphate (NADPH) oxidase [5]. Further, the NADPH oxidase-dependent ROS production is directly proportional to accumulated body fat, although the mechanisms behind this are still not entirely clear [6,7].

In healthy cells, ROS does not implicate harmful effects per se; when consistently regulated, ROS has an intracellular signaling role [8]. This vital balance can be disrupted by an excess of ROS and/or lack of antioxidants (AO) leading to cytotoxic and genotoxic oxidative stress, resulting in DNA strand breaks [8]. The ROS-induced spontaneous deamination of cytosine to uracil (unmethylated cytosine) and 5-hydroxyuracil (methylated cytosine) results in a G:C to A:T transition mutation, since they both preferentially pair with adenosine during DNA replication [9]. Likewise, guanine can be oxidized to 8-oxo-7,8 dihydroguanine (8-oxoG) and leads to a G:C to T:A transition mutation, due to its mispairing with adenosine [9].

In order to ensure DNA integrity, especially in diabetic patients, DNA repair enzymes are crucial. The mismatch repair (MMR) enzyme complex (MLH1 and MSH2 appear to play a key role) acts as a *proofreading* system during DNA synthesis and repairs 8-oxoG lesions [10,11]. MGMT removes alkyl adducts from the DNA especially O^6^-methylguanine, which is read as an adenine by the DNA polymerase. Evidence shows that AO are able to increase DNA repair enzyme activities [12,13]. *MLH1* and *MGMT* promoter are inactivated by hypermethylation, and a promoter methylation-dependent downregulation of the corresponding gene expression in some cancer tissues has been found [14].

Environmental factors have a significant impact upon the epigenetic program of gene expression. Dietary factors have been found to alter DNA methylation both globally, and locus-specifically, leading to a changed expression of genes [15-17]. These modifications can be epigenetically inherited through DNA methylation to the next generation [18].

Numerous epidemiological studies have shown that nutritional folate, by providing one-carbon units, and AO play an important role for both DNA methylation and nucleotide biosynthesis reactions [19,20], and as a consequence, for DNA repair. Folic acid deficiency causes epigenetic changes by diminishing remethylation of s-adenosyl-homocysteine (SAH) to s-adenosylmethionine (SAM) in the methionine cycle, which causes cytosine demethylation, global DNA hypomethylation, and chromosomal instability [21]. In addition, inadequate dietary folate increases uracil misincorporation, rate of DNA strand breaks, and chromosome breaks. Furthermore, folic acid deficiency affects DNA repair by the inhibition of thymidine and purine biosynthesis [21].

Therefore, our primary aim was to assess the impact of the antioxidant- and vitamin-rich diet on the epigenetic pattern of *MLH1* in non-insulin-dependent T2DM (NIDDM2) and patients with impaired fasting glucose (IFG). In this study, we show that a changed diet rich in AO and vitamins (especially folate) has the ability to alter DNA methylation, and compensate ROS-induced epigenetic lesions.

### Study design

This is a post-hoc analysis of a subgroup (n = 15) of a randomized trial (DIAPLANT) conducted at the Department of Nutritional Sciences at the University of Vienna between January and June 2010. We analyzed the DNA methylation of *MLH1*, *MGMT*, and *MSH2* at baseline and after the 8 weeks ongoing intervention in patients with NIDDM2 and IFG (Table 1). To assess the natural and methodological variability of DNA methylation a lean control group (LC) consisting of 8 volunteer, healthy adults, who did not receive the intervention was analyzed separately.

**Table 1:** **Characteristics of the studypopulation**

	**NIDDM2**	**IFG**	**LC**
Number	10	5	8
Women	4	3	5
Age, years	66.30 ± 5.89	69.0 ± 2.35	26.50 ± 1.77
BMI, kg/m^2^	33.8 ± 6.46	27.7 ± 4.14	21.2 ± 1.55

Results for age and BMI presented as mean ± SD. Number, number of participants; BMI, body mass index; NIDDM2, patients with non-insuline-dependent diabetes mellitus type 2; IFG, patients with impaired fasting glucose; LC, lean control group.

Briefly, the aim of the DIAPLANT study was to investigate the positive effects of a dietary intervention with 300 g vegetables and 25 ml walnut oil per day, comprising approximately 73% polyunsaturated fatty acids (PUFA) and 18% monounsaturated fatty acids (MUFA), for 8 weeks, on the risk factors for late diabetic complications in T2DM subjects. The focus was on antioxidant and vitamins (especially folate), and rich green vegetables (broccoli, zucchini, brussels sprouts, green beans, cabbage turnip, maize, carrots, peas, cauliflower, soya beans, cos lettuce and spinach).

Subjects were recruited from a local diabetic clinic (Diabetes outpatient Clinic, Health Centre South, Vienna, Austria) during their annual health assessment. All subjects receiving the intervention had to have stable metabolic control (constant medication regarding glucose, lipid and uric acid metabolism), glycated hemoglobin (HbA1c) concentration < 9.5%, serum total cholesterol (TC) < 300 mg/dl (< 7.76 mmol/l), serum triglycerides (TG) < 500 mg/dl (< 5.7 mmol/l) and serum creatinine < 2.5 mg/dl (< 221 μmol/l). Only subjects with stable body weight, constant dietary habits and physical activity levels for at least four weeks before entry to the study were included. Subjects who intended to change dietary habits, frequency of physical activity or body weight within the study period were not allowed to participate. Exclusion criteria also included smoking, intake of fish oil capsules and other fatty acids. All medical therapies of subjects were continued unchanged throughout the study. Results of the DIAPLANT study will be reported elsewhere. The study protocol was approved by the Ethical Committee of the City of Vienna (EK09-218-VK_NZ). All participants gave their written consent.

## Results

### Oral folate intake

The calculated mean ± SD of folate contained in the intervention vegetables was 153.00 μg ± 82.32 μg per 300 g. Oral folate intake, assessed by 24-h recalls, was significantly increased after the intervention compared to baseline values (127.66 μg ± 195.27, *P* = 0.024).

### Analysis of *MLH1* methylation

The *Bst*UI restriction site (CGCG) in the *MLH1* promoter analyzed by combined bisulfite restriction analysis (COBRA) was hypomethylated at all time points and possible minor methylation shifts were not detectable (see Additional file 1) [22]. DNA methylation differences between baseline (T0) and after 8 weeks of the intervention (T1) were not significantly different in the intervention groups.

#### Pyrosequencing of the MLH1 region1 (MLH1-1)

**Figure 1:**
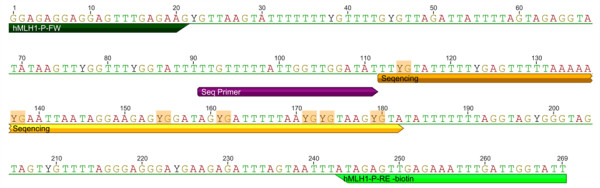
**MLH1 Region 1 sequenceand primers.** MLH1 region 1: Bisulfite-converted *MLH1* promoter 5′-3′sequence and primers used for pyrosequencing. Analyzed CpGs within the sequenced area are shaded orange.

**Figure 2:**
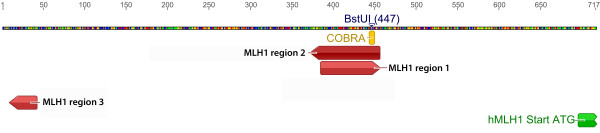
**Analyzed regions within theMLH1 promoter.** CpG methylation assay overview within the *MLH1* promoter region 5^′^- 3^′^. Approximately 730 to 240 bp upstream of the translational start site, 25 CpG sites were analyzed on forward and/or reverse strand.

**Figure 3:**
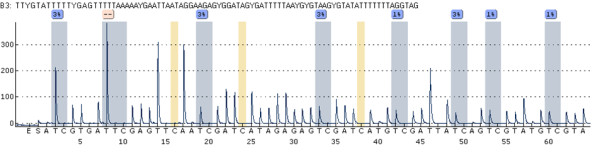
**MLH1 region 1 pyrogram.** MLH1-1 assay showing seven analyzed sites (sample P44 T0). CpG number 2 was not considered for analysis. Three bisulfite treatment controls (16, 24, 38) confirm the complete conversion of unmethylated (non-CpG motif) cytosine residues.

**Figure 4:**
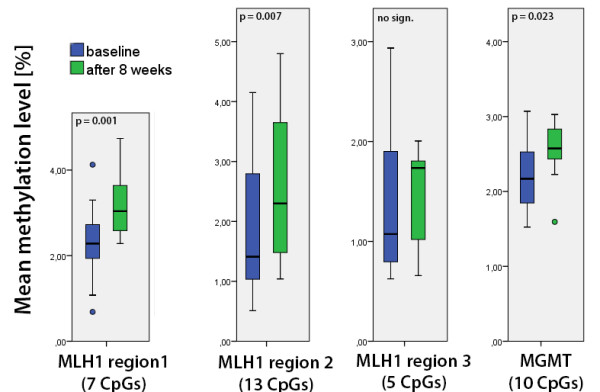
**Mean DNA methylation levels.** Mean DNA methylation levels in the intervention groups. Non-insulin-dependent diabetes mellitus type 2 (NIDDM2) and impaired fasting glucose (IFG) groups were pooled as they showed almost the same picture and no group-specific DNA methylation differences between baseline and T1 were found. *P*-values are based on the paired *t*-test.

**Figure 5:**
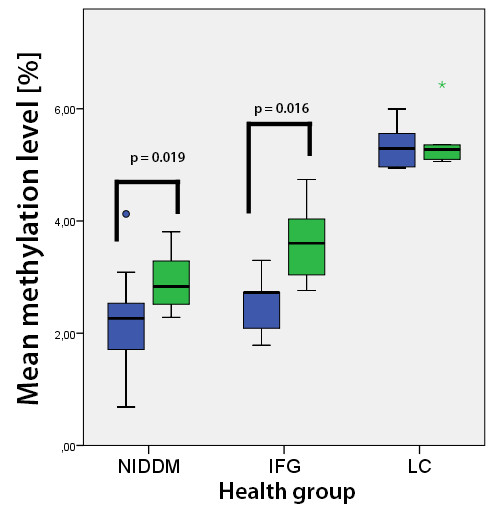
**MLH1 region 1 meanDNA methylation levels.** Detail of MLH1 region 1. Mean DNA methylation levels over seven CpGs in the intervention groups, non-insulin-dependent diabetes mellitus type 2 (NIDDM2) and impaired fasting glucose (IFG), and the lean control group (LC). *P*-values are based on the paired *t*-test.

Quantitative analysis of the investigated area (65 bp, Figure 1 and Figure 2) on the forward strand showed significant changes over time at CpG sites numbers 1, 4 and 6 (Table 2). Site number 2 was not analyzed due to the long 5-nucleotide homopolymer T stretch (Figure 3), which has also been observed to cause problems in analysis, as in other studies [23]. The mean methylation over the seven CpGs was significantly (*P* = 0.001) elevated after the intervention (Figures 4 and 5). This area includes a sequence motif previously analyzed by COBRA [24] at CpG numbers 6 and 7 that has not yet been analyzed by pyrosequencing.

**Table 2:** **MLH1 region 1 group-specificmethylation levels for eachCpG, mean methylation overthe seven CpGs, andpaired*t*-test of methylation changesfrom baseline to eightweeks**

**Health group**		**CpG 1**	**CpG 3**	**CpG 4**	**CpG 5**	**CpG 6**	**CpG 7**	**CpG 8**	**Mean**
NIDDM2		0,666*	0,195	0,810*	−0,025	1,759*	0,032	1,660	0,728*
	SE	0,250	0,429	0,295	0,388	0,539	0,320	1,086	0,254
	*P*	0,026	0,661	0,023	0,950	0,010	0,924	0,161	0,019
IFG		1,250	0,919	1,202	0,466	0,547	2,028	1,372	1,112*
	SE	0,502	0,502	0,740	0,424	0,786	1,828	0,898	0,278
	*P*	0,068	0,141	0,180	0,333	0,525	0,330	0,201	0,016
LC		−0,138	−0,060	−0,165	0,173	0,498	−0,064	0,401	0,092
	SE	0,180	0,322	0,190	0,251	0,227	0,186	0,285	0,184
	*P*	0,487	0,861	0,433	0,529	0,093	0,748	0,232	0,642

CpG numbers 1, 4, 6, and the mean methylation were significantly higher methylated in the IDDM2 group. The IFG group had significantly higher overall methylation. Methylation level remained stable in the LC group. Asterisks indicate significances at *P* < 0.05. NIDDM2, patients with non-insulin-dependent diabetes mellitus type 2; IFG, patients with impaired fasting glucose; LC, lean control group; SE, standard error of the mean.

#### Pyrosequencing of the MLH1 region2 (MLH1-2)

**Figure 6:**
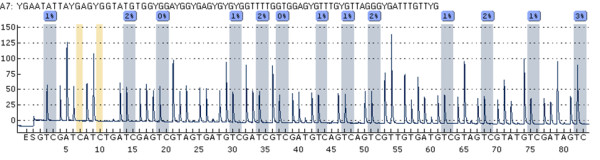
**MLH1 region 2 pyrogram.** The extended MLH1-2 assay pyrogram showing 13 analyzed sites (sample P27 T0).

This region covers a part of the reverse strand of the MLH1-1 region (see Figures 2 and 6.). The mean methylation was significantly higher (0.564% ± 0.696, *P* = 0.007) after the intervention in both NIDDM2 and IFG subjects (Figure 4).

#### Pyrosequencing of the MLH1 region 3 (MLH1-3)

This region is situated 300 bp upstream of the other two MLH1 pyrosequenced regions (see Figure 2.). The methylation levels were very low (0% to 2%) and remained stable over time (Figure 4). No group-specific pattern could be found.

### Analysis of *MGMT* methylation

The *MGMT* mean promoter (see Additional file 2) methylation was significantly higher (0.337% ± 0.468, *P* = 0.023) after the intervention (Figure 4 and Table 3). The percentages of methylated cytosine were generally low (≤ 5%). No correlation of CpG methylation with the *MLH1* expression could be found. DNA methylation differences between baseline and T1 were not significantly different in the intervention groups. Patient number 76 (IFG) showed a baseline methylation level of 11% and 2% after intervention at CpG number 8, leading to a distorted statistic at this position. Excluding patient 76 from pooled statistics leads to + 0.733% overall methylation (SE = 0.146, *P* < 0.001). A European Molecular Biology Open Software Suite (EMBOSS) transcription factor prediction within shared motifs (> 5 bp; 5′-AGCCCG-3′ and 5′-GGACAGC-3′) with *MLH1* region 1 was negative.

**Table 3:** **MGMT group-specific methylation levelsfor each CpG andthe mean methylation overthe ten CpGs**

**Health group**		**CpG 1**	**CpG 2**	**CpG 3**	**CpG 4**	**CpG 5**	**CpG 6**	**CpG 7**	**CpG 8**	**CpG 9**	**CpG 10**	**Mean**
NIDDM2		0,224	0,565	0,272	0,232	0,413	0,436	0,106	0,373*	0,103	0,853	0,420
	SE	0,163	0,270	0,127	0,165	0,191	0,173	0,139	0,155	0,142	0,426	0,192
	*P*	0,210	0,075	0,070	0,202	0,067	0,039	0,468	0,047	0,492	0,086	0,065
IFG		0,226	0,386	0,086	0,183	0,493	0,422	0,129	−1,599	0,168	0,856	0,204
	SE	0,143	0,310	0,088	0,125	0,224	0,214	0,158	1,840	0,255	0,310	0,146
	*P*	0,188	0,281	0,384	0,218	0,092	0,120	0,461	0,434	0,546	0,051	0,233
pooled		0,225	0,496*	0,200*	0,213	0,443*	0,431*	0,115	−0,386	0,128	0,854*	0,337*
	SE	0,110	0,198	0,087	0,109	0,140	0,129	0,101	0,721	0,125	0,279	0,130
	*P*	0,063	0,027	0,039	0,073	0,008	0,006	0,275	0,602	0,327	0,010	0,023

Paired *t*-test of methylation changes between baseline (T0) and 8 weeks (T1). CpG numbers 2, 3, 5, 6, and 10 and the mean methylation were significantly higher methylated in the intervention groups (pooled). Asterisks indicate significances at *P* < 0.05. NIDDM2, patients with non-insulin-dependent diabetes mellitus type 2; IFG, patients with impaired fasting glucose; LC, lean control group; SE, standard error of the mean.

Quantitative analysis of *MSH2* promoter (see Additional file 3) methylation did not reveal any aberrant methylation in the investigated samples. The percentages of methylated cytosine were generally low (≤ 5%). The methylation level remained stable over time. Neither a group-specific pattern nor a correlation of CpG methylation with the *MLH1* expression could be found.

### *MLH1* and *DNMT1* m-RNA expression

Changes in *MLH1* and *DNMT1* gene expression in response to the intervention were quantified by quantitative real-time reverse-transcriptase (qRT)-PCR. Melting curve analysis showed specific product peaks at 87.26°C, 89.34°C, and 83.67°C for *GAPDH*, *DNMT1*, and *MLH1*, respectively. Neither a significant expression shift between the two time points, nor a correlation with methylation levels of CpGs or DNA damage could be found. *MLH1* and *DNMT1* expressions were not affected by the intervention (*P* = 0.306 and *P* = 0.932, respectively).

### DNA damage

**Figure 7:**
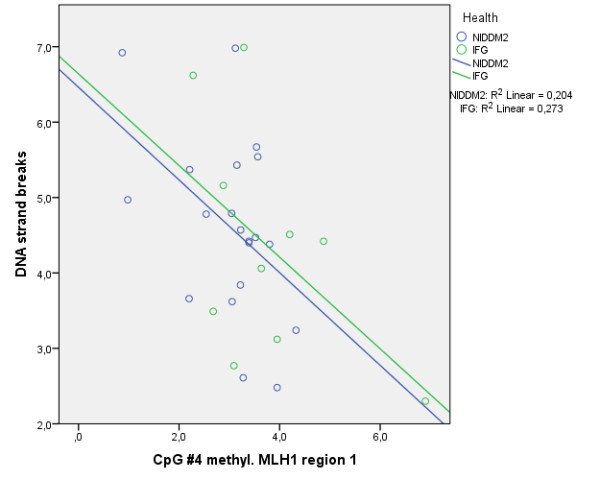
**Correlation between DNA strandbreaks and CpG number4 methylation.** Significant correlation over all time points between the occurrence of DNA strand breaks and the DNA methylation level at CpG number 4 within the MLH1 region 1 (*P* < 0.01; *r* = −0.486).

Baseline levels of DNA strand breaks were comparable between the intervention groups. After the intervention (T1), more DNA strand breaks were measured in the IFG group by the H_2_O_2_-induced oxidative DNA damage test (*P* = 0.006) compared to the NIDDM2 group. We found significant correlations between the occurrence of DNA strand breaks and the methylation level of CpG number 1 (*P* < 0.01; *r* = −0.471; see Figure 7), number 3 (*P* < 0.05; *r* = −0.370), and number 4 (*P* < 0.01; *r* = −0.486) as well as the mean methylation (*P* = 0.05; *r* = −0.361) within the MLH1-1 assay in the NIDDM2 and IFG group at both time points (Figure 4, and Additional files 4 and 5).

## Discussion

While several studies have found that higher ROS levels induce aberrant DNA hypomethylation [25,26], this study is among the first to report that a changed diet can reverse this effect. Our DNA methylation and gene expression studies reveal a link between specific *MLH1* promoter CpG methylations and an optimized diet in IFG and NIDDM2 patients, but no group-specific DNA methylation differences between baseline and T1 could be found. A novel finding is the identification of three variable CpG methylation positions situated in the *MLH1* promoter, which to our knowledge has not been published before. Due to the AO- and folate-rich dietary intervention, the methylation levels of these CpGs were higher compared to the baseline. Our analysis was not intended to show differences in methylation variability between NIDDM2/IFG patients and lean control subjects. Nevertheless, there is clear evidence of a negative correlation between methylation levels and age in our study population.

Chang *et al*. demonstrated that non-cytotoxic levels of H_2_O_2_ significantly reduced the activity of the MMR system in repairing single-base and insertion/deletion loop mismatches in a dose-dependent manner. The different MMR activity was not a result of altered expression levels, but based on posttranslational enzyme degradations by H_2_O_2_. They further propose that ROS-induced stress reduces MMR function and may play a role in the low frequency of microsatellite instability (MSI) detected in inflamed tissues [27]. Therefore, our gene expression data may not reflect MMR activity. Nevertheless, we showed that the variable *MLH1* promoter methylation levels are not affecting its expression, hence not affecting MMR activity.

The detected *MLH1* methylation levels were consistently low (0% to 12%), independently from the time point. Contrary to findings in cancer studies [28], the expression of *MLH1* was not affected by the methylation levels. This suggests that the expression is not regulated by the methylation levels of the analyzed sites in non-cancer patients. Consistent with other findings [29,30], increases in methylation are not explained by higher *DNMT1* expressions. Although the oral intake of folate had increased significantly after the intervention, it had no effect on the *DNMT1* expression. This may indicate that no folate- or methyldonor-deficiency was present before the intervention [31].

Possible histone modification involved in the regulation of *MLH1* expression and its effect on DNA methylation [32-34] do not offer explanations for our results. Histone modifications such as H3K9me3, H3K27me2, and H3K27me3, apparently do not directly affect *MLH1* expression but may serve to index the promoter region for additional epigenetic control [35]. We cannot rule out the involvement of histone modifications leading to higher methylation levels, but in stark contrast we hypothesize that the intervention did not directly induce higher methylation levels, but suppresses the evident loss of methylation before the intervention. CpG-relevant ROS-induced DNA damages are 8-oxoguanine and the conversion of 5-methyl-cytosine (5mC) to 5-hydroxymethylcytosine (5hmC) and 5-hydroxymethyluracil (5hmU), predominantly repaired through the MMR, base excision repair (BER), and nucleotide excision repair (NER) pathway, respectively [36,37]. So far it remains unclear how exactly CpG methylation marks are removed from the DNA, even though active mechanisms have been discussed [38]. Evidence is provided for both direct and indirect demethylation [39,40]. Barreto *et al*. propose that active and direct DNA demethylation is accomplished through a GADD45-dependent process of DNA repair (NER and BER) that involves nucleotide exchange, replacing 5-methyl-cytosine with unmodified cytosine, and it is possible that this is the physiological mechanism that operates typically *in vivo*. *In vitro,* GADD45 knockdown resulted in similar methylation differences to our results (see Barreto *et al*. Figure 3c-d) [39]. Different enzymes (for example TET, AID/APOBEC) have been found to modify the methylated cytosine (by hydroxylation, deamination, oxidation, or a combination of these modifications), leading to its replacement by DNA repair. They act indirectly to mediate DNA demethylation. TET1, 2, and 3 catalyze the conversion of 5mC to 5hmC, which is then replaced with an unmethylated cytosine by the BER enzymes via DNA repair [41].

## Conclusions

Based on these findings we propose that the low initial methylation levels on *MLH1* and *MGMT* are the result of increased oxidative stress, which causes DNA lesions resulting in increased DNA repair activity. The latter leads to a loss of methylation marks by direct and/or indirect demethylation. The nutritional intervention might have led to a lower ROS burden and diminished the demethylating effects, resulting in higher overall methylation levels, and helped to maintain crucial and tissue-specific methylation marks with possible regulatory function. This finding need to be further investigated to reveal the exact underlying mechanisms behind the ROS-induced demethylation in relation to MSI and cancer-relevant promoter demethylation of oncogene, leading to initiation of tumors.

## Methods

### Sample collection

Blood was sampled before (T0) and after 8 weeks of dietary intervention (T1) using PAXgene Blood DNA tubes and the PAXgene Blood RNA tubes (Qiagen, Hilden, Germany). The samples were stored until analyses at −20°C.

### Folate quantification

On the day before blood sampling, 24-h recalls of food intake were obtained from the subjects. The dietary composition of the vegetables and 24-h recalls were evaluated using the nutritional software NUT.S (BLS II.3.1., Karlsruhe, Germany).

### DNA isolation and bisulfite conversion

DNA was extracted using the PAXgene Blood DNA Kit (Qiagen, Hilden, Germany) according to the manufacturer’s instructions. For methylation analysis, all samples were bisulfite-converted with the EpiTectBisulfite Kit (Qiagen, Hilden, Germany), resulting in the deamination of unmethylated cytosine to uracil, whereas methylated cytosine remain unchanged. The EpiTect Whole Bisulfitome Kit (Qiagen, Hilden, Germany) was used to amplify 2 μl converted DNA. The concentration of DNA was measured on a Pico100 (Picodrop Limited, Hinxton, UK). All reactions were carried out according to manufacturer’s protocols. Samples were stored at −20°C.

### RNA isolation and reverse transcription

Total RNA was extracted from blood samples using the PAXgene Blood RNA Kit (Qiagen, Hilden, Germany) following the producer’s handbook. The random primers (hexamers) of the Phusion RT-PCR Kit (Finnzymes, Vantaa, Finland) were used to reverse-transcribe 5 μl of total RNA into single-stranded DNA. The concentration of cDNA was measured on a Pico100 (Picodrop Limited, Hinxton, UK). The samples were stored at −20°C.

### PCR and restriction enzymatic digestion - COBRA

All bisulfite-treated samples were investigated by PCR and endonuclease digestion. The primers of Deng *et al*. were used for PCR [24]. The two methylation-specific primers MLH1-C-FW and MLH1-C-RE (Table 4) were chosen for the touchdown PCR amplification. The PCR was always carried out in 50 μl reaction mixtures; these contained 25 μl 2× SensiMix Probe polymerase mastermix (Bioline, London, UK), 625 nM of each primer, and 20 ng bisulfite-converted DNA. PCR conditions were 95°C for 10 minutes, 15 cycles of 95°C for 10 s, 55°C to 51°C for 1 minute, -0.2°C per cycle, 72°C for 1 minute followed by 25 cycles of 95°C for 40 s, 51°C for 40 s, 72°C for 40 s and a final elongation at 72°C for 10 minutes. After amplification, the PCR products were purified by isopropanol-precipitation. For enzymatic digestion, 10 μl PCR product was digested with 1U of BstUI (New England Biolabs, Frankfurt am Main, Germany) and NEB4 Buffer in 25 μl total volume at 60°C for 3 h. The digested fragments were separated by 2% agarose gel electrophoresis. Both CpG sites within the BstUI need to be methylated to be digested by BstUI. If the recognition site (CGCG) is methylated, its ssPCR product (294 bp) is digested into a 206 bp and an 88 bp fragment. If the CGCG motif is not methylated, the amplicon is not digested. The digested DNA was separated on 3.5% agarose gels in 1× tris-acetate ethylenediaminetetraacetic acid (TAE) and stained with GelRed (Biotium, Hayward, CA). Bands were analyzed using ImageJ 1.44p and quantified relative to the synthetic fully methylated /unmethylated control-DNA (Qiagen, Hilden, Germany). The proportion of methylated versus unmethylated DNA was determined from the relative intensities of cut and uncut PCR product.

**Table 4:** **Primers used for PCRmethods**

**Primer name**	**Sequence 5′-3′**	**Application**	**Size**	**Number of CpGs**	**Ref**
MLH1-C-FW	GTATTTTTGTTTTTATTGGTTGGATA	COBRA	294 bp	1	[24]
MLH1-C-RE	AATACCTTCAACCAATCACCTCAATA	COBRA			
MLH1-1-FW	GGAGAGGAGGAGTTTGAGAAG	PSQ	269 bp	7	
MLH1-1-RE	Biotin-AATACCAATCAAATTTCTCAACTCTAT	PSQ			
MLH1-1-Seq	TTGTTTTTATTGGTTGGATAT	PSQ			
MGMT-FW	Biotin-GGATATGTTGGGATAGTT	PSQ	266 bp	5	[42]
MGMT-RE	AAACTAAACAACACCTAAA	PSQ			
MGMT-Seq	CCCAAACACTCACCAAA	PSQ			
MLH1-Q-FW	TTCTCAGGTTATCGGAGCCAGCAC	qRT-PCR	288 bp		[43]
MLH1-Q-RE	CTTCGTCCCAATTCACCTCAGTGG	qRT-PCR			
DNMT1-Q-FW	ACCGCTTCTACTTCCTCGAGGCCTA	qRT-PCR	335 bp		[44]
DNMT1-Q-RE	GTTGCAGTCCTCTGTGAACACTGTGG	qRT-PCR			
GAPDH-Q-FW	CGACCACTTTGTCAAGCTCA	qRT-PCR	205 bp		[45]
GAPDH-Q-RE	AGGGGTCTACATGGCAACTG	qRT-PCR			

COBRA, combined bisulfite restriction analysis: PSQ, pyrosequencing: qRT-PCR, quantitative real-time reverse-transcriptase polymerase chain reaction; Ref, reference.

### Quantitative promoter methylation analyses by DNA pyrosequencing

We performed quantitative methylation analyses of the *MLH1*, *MGMT*, and *MSH2* promoter by pyrosequencing of sodium bisulfite-modified DNA using the PyroMark Q24 (Qiagen, Hilden, Germany). DNA methylation was determined in three different regions inside the *MLH1* promoter (Figure 2). PCR were performed in 25 μl reaction mixtures with 12.5 μl PyroMark 2× PCR master mix, 280 nM of each primer (Table 4), 2.5 ml CoralLoad Concentrate 10× (Qiagen, Hilden, Germany), and 25 ng bisulfite converted DNA for *MGMT* and 25 ng of EpiTect Whole Bisulfitome (Qiagen, Hilden, Germany)-amplified DNA for all other analyses. PCR conditions were 95°C for 15 minutes; 45 cycles of 94°C for 30 s, a.) for *MGMT* 47.5°C for 45 s, b.), for all other genes, 56°C for 30 s; 72°C for 30 s, and a final elongation at 72°C for 10 minutes. One of the PCR primers was biotinylated to purify the final PCR product using streptavidin-coated Sepharose® beads (GE Healthcare, Vienna, Austria). Additionally three PyroMark CpG assay kits (Qiagen, Hilden, Germany) were used following the manufacturer’s instructions (Table 5). The MLH1-2 assay was manually extended to analyze thirteen CpGs instead of six. PCR products were checked by 3.5% agarose gel electrophoresis. A total of 12.5 μl of the PCR product was used for subsequent pyrosequencing using a PyroMark Q24 System (Qiagen, Hilden, Germany). Briefly, the sepharose beads containing the immobilized PCR product were washed and denatured using 0.2 M NaOH solution and rewashed using the pyrosequencing Vacuum Prep Tool (Qiagen, Hilden, Germany). The purified single-stranded PCR product was released to the annealing buffer (Qiagen, Hilden, Germany) containing the corresponding pyrosequencing primer (300 nM). Subsequent quantification of CpG methylation levels was performed using the PyroMark Q24 software (Qiagen, Hilden, Germany). Each pyrosequencing assay was performed on duplicates. For quality control, each experiment included non-CpG cytosines as internal controls to verify efficient sodium bisulfite DNA conversion. We also included synthetic fully methylated /unmethylated control-DNA (Qiagen, Hilden, Germany).

**Table 5:** **PyroMark CpG assay kits(Qiagen, Hilden, Germany)**

**Assay name**	**GeneGlobe Catalogue number**	**Size**	**Sequence to analyze 5′-3′**	**Number of CpGs**
MLH1-2	PM00104860	203 bp	YGAATATTAYGAGYGGTATGTGGYGGAYGGYGA	6 (13)*
MLH1-3	PM00104839	246 bp	GYGTTTGYGYGTTAGAGATYGTTGTTYGT	5
MSH2	PM00007777	190 bp	YGYGTTTGYGGGTTTTYGYGYGATTTAGGYGT	7

* provided kit was manually extended to analyze 13 instead of 6 CpGs.

### qRT-PCR

The mRNA level of *MLH1*, *DNMT1*, and *GAPDH* as an endogenous control gene for normalization was analyzed in triplicates on a StepOne Plus real-time PCR system (Applied Biosystems, Vienna, Austria) using the SYBR Green approach. Each reaction mix consisted of 5 μl SensiFAST SYBRgreen (Bioline GmbH, London, UK), 300nM primers (Table 4), and 100 ng cDNA to a total volume of 10 μl. PCR conditions were 95°C for 2 minutes, 40 cycles of 95°C for 30 s, 60°C for 10 s, and 72°C for 5 s, followed by a melt curve analysis. The efficiency for *DNMT1*, *MLH1*, and *GAPDH*, was 103.5%, 106.7%, and 109.4%, respectively. Calculations were performed using the comparative Ct method.

### Comet assay

A Comet assay was performed based on the protocol published by Azqueta [46]. For the evaluation of DNA damage, Komet 5.5 image analysis software (Kinetic Imaging Limited, Nottingham, UK) was used, which was linked to a fluorescent microscope. For every sample two gels with 50 cells each were randomly scored. The percentage of DNA in the tail (% tail DNA) was determined and the mean was calculated.

### Statistical analyses

Methylation levels and expression data of 23 paired samples were analyzed with SPSS 20 (IBM, Armonk, NY). The Kolmogorov-Smirnov test was used to test for normality of the distributions. The Student’s two-tailed paired *t*-test and one-way analysis of variance (ANOVA) with the Scheffé post hoc correction test were used to determine significant differences. The two-tailed Pearson test was used to determine correlations between methylation level, gene expression, and amount of DNA strand break. All statistics were tested for possible associations with age and sex; no significant associations were found. A *P*-value < 0.05 was considered statistically significant. All data shown are mean ± SD unless otherwise indicated.

## Abbreviations

ANOVA: one-way analysis of variance; AO: antioxidants; BER: base excision repair; COBRA: combined bisulfite restriction analysis; DHA: docosahexaenoic acid; HbA1c: glycated hemoglobin; IFG: impaired fasting glucose; MMR: mismatch repair; MSI: microsatellite instability; MUFA: monounsaturated fatty acids; NADPH: nicotinamide adenine dinucleotide phosphate; NER: nucleotide excision repair; 8-oxoG: 8-oxo-7,8 dihydroguanine; PSQ: pyrosequencing; PUFA: polyunsaturated fatty acids; qRT-PCR: quantitative real-time reverse-transcriptase polymerase chain reaction; ROS: reactive oxygen species; SAH: s-adenosyl-homocysteine; SAM: s-adenosylmethionine; TAE: tris-acetate ethylenediaminetetraacetic acid; TC: total cholesterol; T2DM: diabetes mellitus type 2; TG: serum triglycerides.

## Competing interests

The authors declare that they have no competing interests.

## Authors' contributions

OJS initiated the study, performed the methylation and expression experiments, analyzed the data, and wrote the manuscript. EM participated in the DIAPLANT study design, performed the COMET assay experiments, and analyzed the data. HB participated in designing the DIAPLANT study, recruited the study subjects and was responsible for their medical treatment. EA participated to the elaboration of the study and experiment design. K-HW participated in designing the DIAPLANT study. AGH conceived and designed the study. All authors read and approved the final manuscript.

## Supplementary Material

Additional file 1: **Figure S1.** COBRA gelelectorphoresis. Figure showing gelelectorphoresis of combined bisufite restriction analysis (COBRA) before (10 μl) and after (10 μl and 15 μl) *BST*UI digestion. 100 bp DNA ladder. (PNG 332 kb) Click here for file

Additional file 2 **Figure S2.** MGMT pyrosequencing location. Figure showing methylation assay overview within the *MGMT* promoter region 5′- 3′. Ten CpG sites were analyzed by reverse-sequencing the upper strand. CpG island concentration is shown in the lower green. (PNG 56 kb) Click here for file

Additional file 3 **Figure S3.** MSH2 pyrosequencing location. Figure showing methylation assay overview within the *MSH2* promoter region 5′- 3′. Approximately 260 to 230 bp upstream of the translational start site, seven CpG sites were analyzed on the forward strand. CpG island concentration is shown in the lower green. (PNG 32 kb) Click here for file

Additional file 4: **Figure S4.** Correlation between DNA strand breaks and CpG number 1 methylation. Figure showing significant correlation over all time points between the occurrence of DNA strand breaks and the DNA methylation level at CpG number 1 within the MLH1 region 1 (*P* <0.01; *r* = −0.471). (PNG 33 kb) Click here for file

Additional file 5 **Figure S5.** Correlation between DNA strand breaks and mean methylation. Figure showing correlation over all time points between the occurrence of DNA strand breaks and the mean DNA methylation level of the MLH1 region 1 (*P* = 0.05; *r* = −0.361). (PNG 31 kb) Click here for file
