# Supplementary figures and images for: Vitamin and antioxidant rich diet increases MLH1 promoter DNA methylation in DMT2 subjects
Source: Clin Epigenetics. 2012 Oct 1;4(1):19. doi: 10.1186/1868-7083-4-19 (PMC3579724; doi:10.1186/1868-7083-4-19)

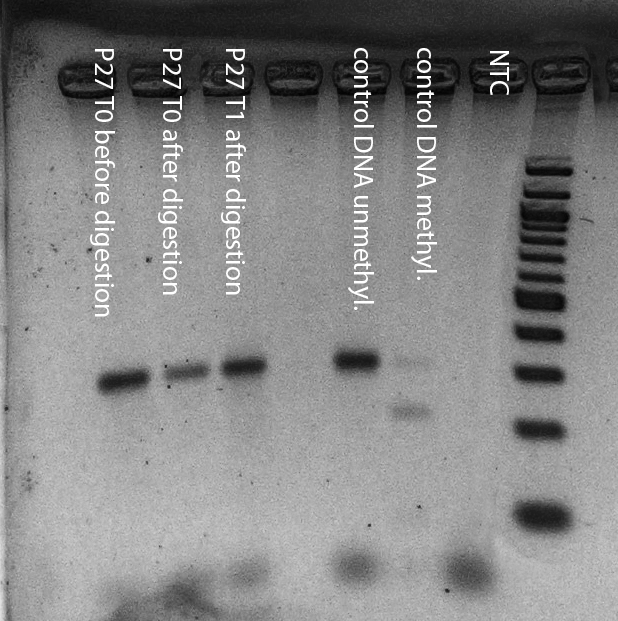

Supplement: Additional file 1: — Figure S1. COBRA gelelectorphoresis. Figure showing gelelectorphoresis of combined bisufite restriction analysis (COBRA) before (10 μl) and after (10 μl and 15 μl) BSTUI digestion. 100 bp DNA ladder. (PNG 332 kb) [file 1868-7083-4-19-S1.png]

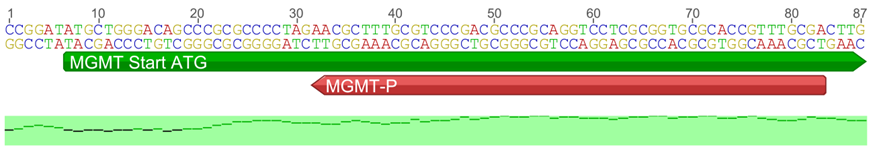

Supplement: Additional file 2 — Figure S2. MGMT pyrosequencing location. Figure showing methylation assay overview within the MGMT promoter region 5′- 3′. Ten CpG sites were analyzed by reverse-sequencing the upper strand. CpG island concentration is shown in the lower green. (PNG 56 kb) [file 1868-7083-4-19-S2.png]

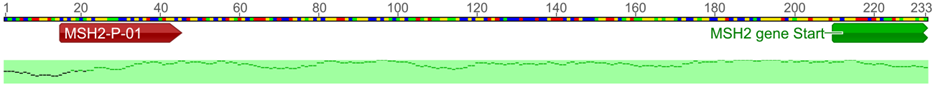

Supplement: Additional file 3 — Figure S3. MSH2 pyrosequencing location. Figure showing methylation assay overview within the MSH2 promoter region 5′- 3′. Approximately 260 to 230 bp upstream of the translational start site, seven CpG sites were analyzed on the forward strand. CpG island concentration is shown in the lower green. (PNG 32 kb) [file 1868-7083-4-19-S3.png]

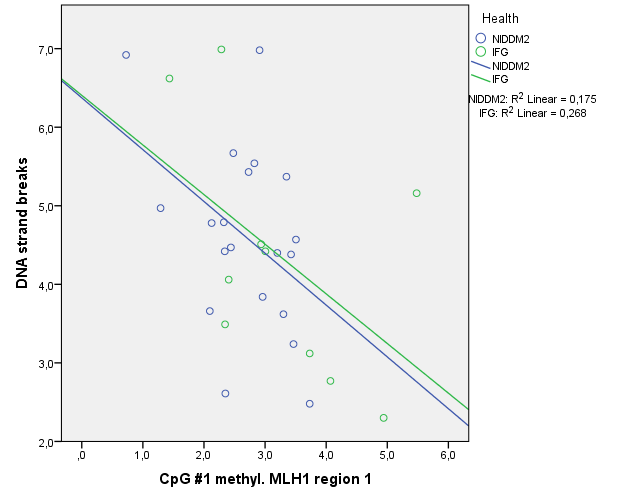

Supplement: Additional file 4: — Figure S4. Correlation between DNA strand breaks and CpG number 1 methylation. Figure showing significant correlation over all time points between the occurrence of DNA strand breaks and the DNA methylation level at CpG number 1 within the MLH1 region 1 (P <0.01; r = −0.471). (PNG 33 kb) [file 1868-7083-4-19-S4.png]

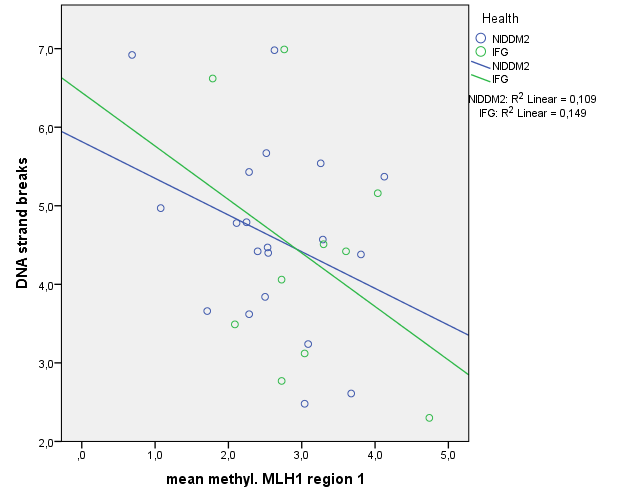

Supplement: Additional file 5 — Figure S5. Correlation between DNA strand breaks and mean methylation. Figure showing correlation over all time points between the occurrence of DNA strand breaks and the mean DNA methylation level of the MLH1 region 1 (P = 0.05; r = −0.361). (PNG 31 kb) [file 1868-7083-4-19-S5.png]
